# Multiple Musculotendinous Variations in the Limbs: A Cadaveric Case Report

**DOI:** 10.7759/cureus.77192

**Published:** 2025-01-09

**Authors:** Nil Kamal Jaber, Camille Charrier, Nemine Maouloud, Oceane Legendre, Femina Sam

**Affiliations:** 1 Human Anatomy Unit, Department of Biomedical Sciences, School of Infection, Inflammation and Immunology, College of Medicine and Health, University of Birmingham, Birmingham, GBR; 2 Faculty of Medicine, University of Tours, Tours, FRA; 3 Department of Anatomy, Christian Medical College, Vellore, IND

**Keywords:** abductor, accessory, dissection, extensor, flexor, forearm, gantzer, variations, wrist

## Abstract

Muscle and tendon variations in the upper and lower extremities are common but often go unnoticed due to their asymptomatic nature, typically being incidental findings on radiological imaging. These anatomical variations, widely documented in scientific literature, can impact clinical procedures and surgeries. Particularly, the accessory muscles have been implicated in different forms of entrapment neuropathy syndromes. This case report details the muscle and tendon variations observed during the short-term dissection project. The variations included additional muscle in the flexor aspect of the proximal right forearm and additional tendinous slips of abductor pollicis longus forming the boundaries of the anatomical snuffbox in the right wrist of the upper limb. Additionally, we report the presence of additional tendinous slips of the extensor hallucis longus in the left lower limb. From a clinical perspective, recognizing these variations is essential for procedures such as tendon transfers, where an accessory muscle or a tendon could either complicate or facilitate the tendon transfers within the anatomical landscape.

## Introduction

Accessory muscles in the limbs frequently cause arterial and nerve entrapments, leading to compression syndromes. Accurate diagnosis of these entrapments, including identifying their precise cause and location, is essential before surgical decompression [1]. While accessory muscles have been widely reported, multiple variations in a single individual are rare. Each variation presents a unique anatomical perspective, particularly to nearby structures. These accessory muscles have been reported in various regions throughout the human body, and some are well known for their high incidence [2]. Knowledge of these muscle variations aids in identifying areas of compression and is a deciding factor for appropriate treatment for compressive syndromes [3].

This report describes the muscular and tendinous variations seen in a cadaver during our short-term dissection project. We report the presence of additional muscle in the flexor aspect of the right forearm, along with the additional tendinous slips forming the boundaries of the anatomical snuffbox. Additionally, we report the presence of additional slips of the extensor hallucis longus in the left lower limb. These muscular variations, though reported previously, show different anatomical configurations.

## Case presentation

During our internship at the Human Anatomy Lab as a part of a dissection project, the following multiple muscular variations were seen in the extremities of a middle-aged cadaver. The donor provided consent for images to be taken as part of the donor registration program at the University of Birmingham, license number 12236. The authors clarify that research involving human cadaveric tissue was conducted under the HTA License. All efforts were made to adhere to ethical guidelines and legal requirements governing the use of human cadaveric donors in anatomical research. Written informed consent was obtained from the donor for their participation in the research.

Accessory muscle deep to the flexor digitorum superficialis in the right forearm

**Figure 1:**
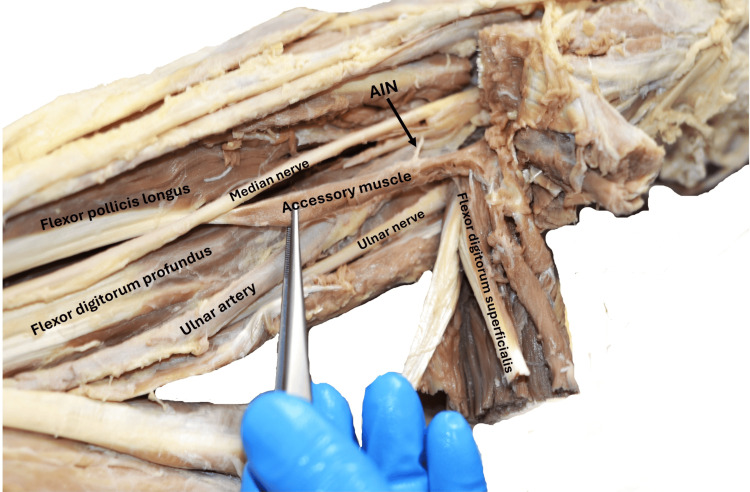
Dissection of the right upper limb showing the proximal part of the forearm The accessory muscle originated from the deep surface of the superficial forearm flexor group of muscles and was inserted into the FPL. The muscle was related superficially to the median nerve, anterior interosseous nerve, and ulnar artery. It was supplied by the AIN. The median nerve emerged from the muscle's deep surface and became superficial at the insertion of the tendon of the accessory muscle into the FPL. (As the superficial flexor muscles have been reflected medially, the relation of the additional muscle to the median nerve couldn’t be shown in the figure described in the report.) FPL: flexor pollicis longus, AIN: anterior interosseous nerve

An accessory muscle originated from the deep surface of the superficial forearm flexor group of muscles, without any bony origin. The muscle was long, slender, and spindle-shaped. Its muscle belly transitioned into a thin tendon as it obliquely crossed the mid-forearm from the medial to the lateral side. The muscle was lying superficial to the median nerve, anterior interosseous nerve, and ulnar artery. The thin tendon of the muscle ran downwards posteriorly to the median nerve at the musculotendinous junction and was attached to the ulnar side of the flexor pollicis longus tendon. The accessory belly, commonly called Gantzer’s muscle, was supplied by one of the muscular branches of the anterior interosseous nerve from its deeper aspect (Figure 1).

Additional tendinous slips bordering the anatomical snuff box

**Figure 2:**
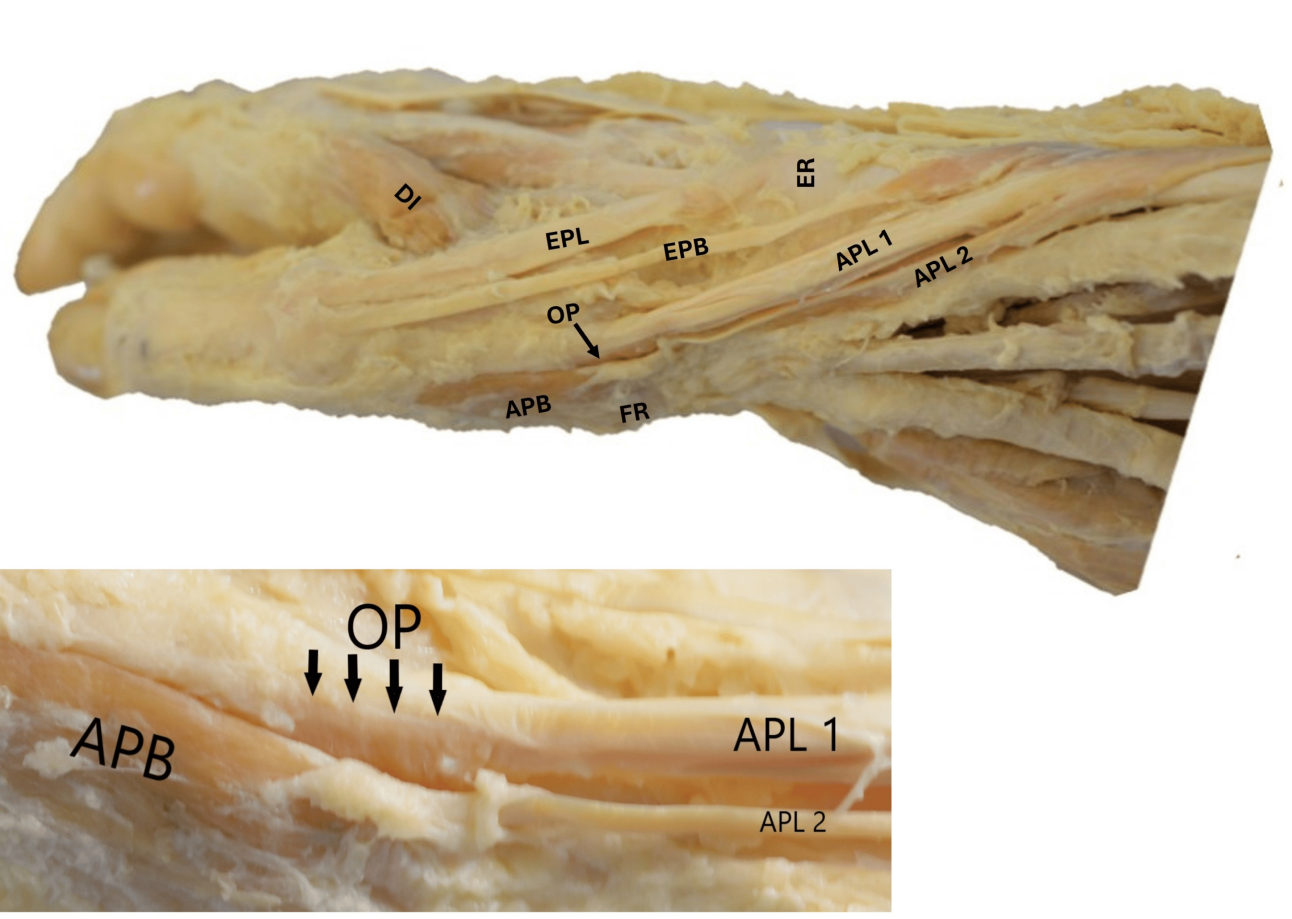
Dissection of the right upper limb showing the distal third of the forearm, wrist, and the dorsolateral aspect of the hand The APL with its two muscle bellies. The tendon of the medial belly (APL 1) was inserted into the radial aspect of the OP muscle and the dorsal aspect of the base of the first metacarpal. The tendon of the lateral belly (APL 2) merged with the APB and FR muscle belly. The magnified image shows the insertion of the APL 1 tendon into the radial aspect of the OP muscle fibers, which is deep into the APB. EPL: extensor pollicis longus, EPB: extensor pollicis brevis, ER: extensor retinaculum, DI: dorsal interossei, APL: abductor pollicis longus, OP: opponens pollicis, APB: abductor pollicis brevis, FR: flexor retinaculum

In the right forearm near the wrist, the abductor pollicis longus had two distinct muscle bellies that originated from the shafts of the radius and ulna, as well as the interosseous membrane. The lateral belly of the abductor pollicis longus formed a thin tendon, which curved around the wrist and merged with the muscle belly of the abductor pollicis brevis and the flexor retinaculum. The medial belly resembled the main abductor pollicis longus. It formed a tendon that was inserted into the radial aspect of the muscle belly of the opponens pollicis, deep to the muscle belly of the abductor pollicis brevis, and the dorsal aspect of the base of the first metacarpal. The other tendons around the anatomical snuffbox showed no variations. The cephalic vein and the superficial branch of the radial nerve passed between the tendons of the lateral belly of the abductor pollicis longus and extensor pollicis brevis on the lateral side, and the tendon of the extensor pollicis longus on the medial side (Figure 2).

Additional tendinous slip of extensor hallucis longus

**Figure 3:**
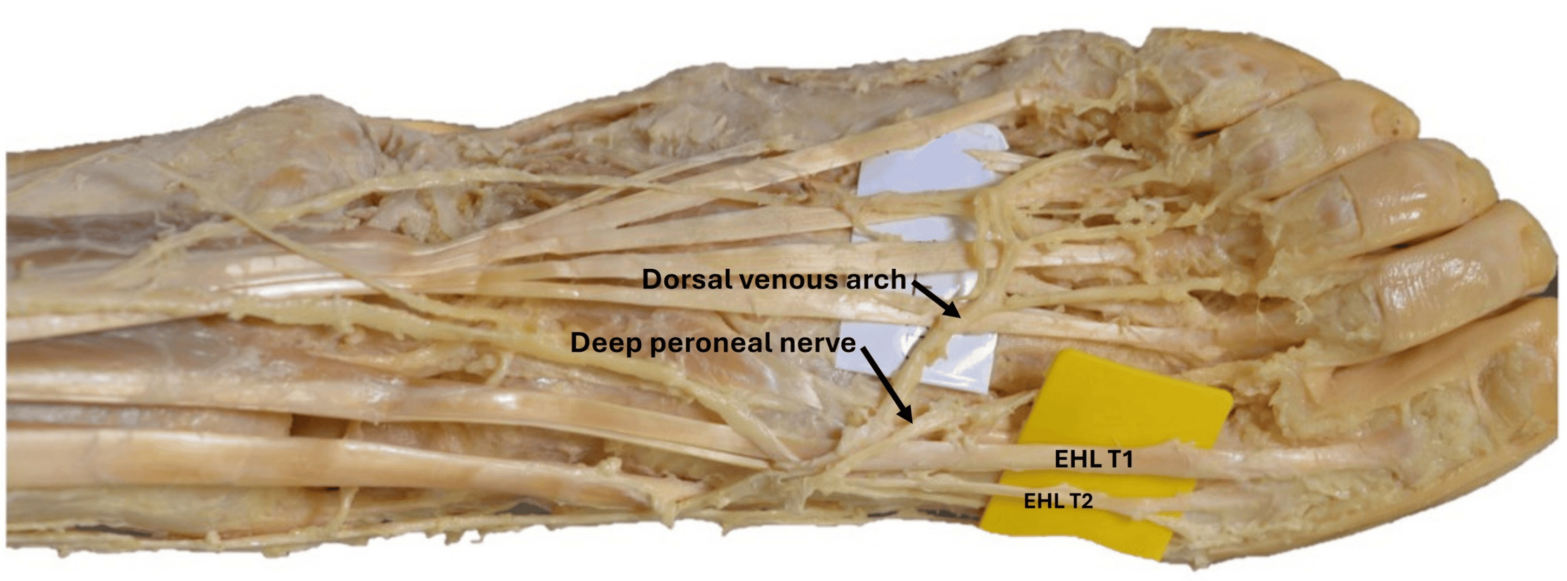
Dissection of the left lower limb showing the distal third of the leg, ankle, and the dorsal aspect of the foot The EHL had two distal tendons. The EHL T1 tendon was inserted into the dorsal aspect of the base of the distal phalanx of the great toe, while the EHL T2 tendon was inserted into the base of the proximal phalanx of the great toe. Note its bifid distal attachment. EHL: extensor hallucis longus

In the left foot, the extensor hallucis longus had two distal tendons. After passing deep to the extensor retinaculum and crossing the ankle joint, the main tendon of the extensor hallucis longus split into two at the base of the first metatarsal bone. The thicker tendon was inserted into the dorsal aspect of the base of the distal phalanx of the great toe, while the thinner tendon had a separate insertion into the base of the proximal phalanx of the great toe, positioned just proximal and medial to the insertion of the thicker tendon. The distal attachment of the thin tendon was bifid. Superficial to these tendons, the dorsal venous arch and cutaneous branches of the deep peroneal nerve were observed (Figure 3).

## Discussion

From the embryological point of view, myotomes, which are derived from somites, produce precursors for the musculoskeletal system. Growth factors in limb buds and adhesion molecules on myoblasts guide myoblast distribution in the limb [4]. The muscle primordia thus formed from different layers fuse to form a single muscle, and some muscle primordia disappear through cell death. The persistence of some muscle primordia or any disruptions in myotome formation or adhesion molecule expression can result in anomalous muscle slips [4].

Among the anatomical variations of the upper limb muscles, Gantzer’s muscle is a relatively new, consistent additional muscle in the flexor compartment of the forearm. It is also named the accessory head of the flexor pollicis longus and the accessory head of the flexor digitorum profundus [5-7]. This muscle belly descends beneath the flexor digitorum superficialis up to the mid-forearm and is present in two-thirds of the population. It originates from the medial epicondyle of the humerus, the coronoid process of the ulna, or the fascial sheath of flexor digitorum superficialis or pronator teres [8]. It inserts onto one of the deep flexors, i.e., flexor pollicis longus and flexor digitorum profundus [6]. The anterior interosseous nerve, a branch of the median nerve, innervates accessory muscle, although some studies have reported innervation directly from the median nerve [6]. In the present report, the variant was inserted into the tendon of the flexor pollicis longus and innervated by the anterior interosseous nerve. The meta-analysis by Asghar in 2022 showed that the prevalence of this accessory FPL type of muscle was around 48% of the general population, and they were present more frequently on the right side (49%) [6]. This accessory muscle was often positioned between the median and anterior interosseous nerve. Thus, it has been assumed as one of the causes of "anterior interosseous nerve syndrome" [5,9]. This disorder also often leads to loss of pinching. In this report, the accessory muscle belly was noticed to be superficial to the median nerve and anterior interosseous nerve and was innervated by the anterior interosseous nerve from its deep surface. The muscle formed a thin tendon near its insertion point and was related deep to the median nerve. While considering its relation to the vascular structures, this accessory muscle directly sits on the ulnar artery and may lead to compression syndromes. The presence of these accessory muscles could potentially alter the development of the neurovascular structures, thus causing variations in the nerve branching points or the artery bifurcation points [5].

Multiple variations of the tendons of the anatomical snuffbox have been discovered through anatomical research. The anatomical snuffbox is bordered by three tendons, which are known to have numerous tendinous slips and different attachment areas. This is clinically significant because it may predispose to the development of tendinopathy [10]. De Quervain tenosynovitis is a stenosing tendinopathy that affects the forearm's first extensor compartment. The presence of multiple tendons, as in this case report, might be a causative factor. As the anatomical snuffbox is a clinically significant area for accessing the cephalic vein and radial artery, these tendinous variations alter the location of the anatomical structures, leading to pseudo-aneurysm, arterial occlusion, or hematoma [11]. Previous studies reported the incidence of two to seven additional slips of abductor pollicis longus [12]. It was categorized into types 1 to 3 with subtypes [13]. In this report, we identified a variation of type 3 that has not previously been classified as a subtype.

In the lower extremities, variations in the extensor hallucis longus have been reported in studies regarding its additional bands and their insertion [14]. There are reports of accessory tendinous slips of the extensor hallucis longus tendon called extensor primi internodii hallucis, extensor hallucis capsularis, and extensor ossis metatarsi hallucis related to the attachment sites [15]. A few authors tried to categorize the variations of the tendons of the extensor hallucis longus. Olewnik et al. and Zielinska et al. proposed a classification system to categorize the variations in the tendons from type 1 to type 3 with various subtypes [14,15]. According to Olewnik et al.'s classification, the tendinous insertion in the present report belongs to type 11b [15].

## Conclusions

Anatomical variations of the musculature and tendons may lead to diagnostic confusion while interpreting radiological images. Knowledge of these additional slips will certainly be useful in interpreting the variations in the radiological images. It also helps surgeons in tendon transfer surgeries to treat traumatic tendon ruptures or corrective surgeries for deformities like hallux varus.
